# Revealing barriers and facilitators to use a new genetic test: comparison of three user involvement methods

**DOI:** 10.1007/s12687-012-0080-6

**Published:** 2012-02-09

**Authors:** Martijn D. F. Rhebergen, Maaike J. Visser, Maarten M. Verberk, Annet F. Lenderink, Frank J. H. van Dijk, Sanja Kezic, Carel T. J. Hulshof

**Affiliations:** 1 Coronel Institute of Occupational Health, Academic Medical Center, University of Amsterdam, P.O. Box: 22700, Meibergdreef 9, 1100 DE Amsterdam, the Netherlands; 2 Dutch Center for Occupational Diseases, Academic Medical Center, University of Amsterdam, P.O. Box: 22660, Tafelbergweg 51, 1100 DD Amsterdam, the Netherlands

**Keywords:** User involvement, Genetic testing, Public opinion, Attitude, Contact dermatitis, Occupational health

## Abstract

We compared three common user involvement methods in revealing barriers and facilitators from intended users that might influence their use of a new genetic test. The study was part of the development of a new genetic test on the susceptibility to hand eczema for nurses. Eighty student nurses participated in five focus groups (*n* = 33), 15 interviews (*n* = 15) or questionnaires (*n* = 32). For each method, data were collected until saturation. We compared the mean number of items and relevant remarks that could influence the use of the genetic test obtained per method, divided by the number of participants in that method. Thematic content analysis was performed using MAXQDA software. The focus groups revealed 30 unique items compared to 29 in the interviews and 21 in the questionnaires. The interviews produced more items and relevant remarks per participant (1.9 and 8.4 pp) than focus groups (0.9 and 4.8 pp) or questionnaires (0.7 and 2.3 pp). All three involvement methods revealed relevant barriers and facilitators to use a new genetic test. Focus groups and interviews revealed substantially more items than questionnaires. Furthermore, this study suggests a preference for the use of interviews because the number of items per participant was higher than for focus groups and questionnaires. This conclusion may be valid for other genetic tests as well.

## Background

Research knowledge reaches healthcare practice only partially and through a process that on average takes many years (Balas and Boren 2000; Glasziou and Haynes 2005). It is a complicated process that requires changes in behaviour, practices and policy from different stakeholders (Straus et al. 2009). An important activity or action before applying a new knowledge product in practice is the identification of items that can hinder or facilitate the use of this product (Graham et al. 2006; Straus et al. 2009). Barriers and facilitators are often related to the research product itself, the context and the implementation strategies used (Greenhalgh et al. 2004; Grol and Wensing 2006). Accounting for these barriers and facilitators prior to actual application is supposed to result in knowledge products that are better tailored to the needs of the intended users and to the context (Graham et al. 2006; Straus et al. 2009; Ward et al. 2009).

The involvement of intended users is recognised to be important for the identification of potential barriers and facilitators (Bartholomew et al. 2006; Graham et al. 2006; Grol and Wensing 2006; International Organization for Standardization (ISO) 1999; Kujala 2003; Steiner and Norman 2008; Straus et al. 2009). The most common methods of involving users are focus groups, interviews and questionnaires (Bryman 2001; Denzin and Lincoln 2000; Kvale 1996). In the social sciences, these three methods are considered to be “qualitative research methods”. The aim of using these methods is to explore the diversity of attitudes, ideas or beliefs on potential barriers and facilitators to use a new knowledge product (Denzin and Lincoln 2000). In general, individual interviews and focus groups are utilised to collect in-depth data on a small number of people, where focus groups are supposed to have the additional advantage that they can encourage discussion between participants when needed. Questionnaires are used to collect less in-depth data on a larger group of individuals. Remarkably, research comparing the output and efficiency of these methods, e.g. the number of barriers and facilitators taking into account the effort to obtain them, is scarce (Morgan 1996).

Involving users and analysing their attitudes, ideas or beliefs takes time and effort. If one method, or a combination of methods, has a higher output per participant, it would be a more attractive option in the process of applying new knowledge products in practice. We used the opportunity to compare three common involvement methods in an ongoing scientific study aiming at developing a genetic test for the susceptibility to hand eczema. Involvement of potential users of this genetic test prior to its application in practice was used to anticipate on its (clinical) utility and on ethical, legal and social issues such as described in the ACCE framework or Evaluation of Genomic Application in Practice and Prevention initiative (Sanderson et al. 2005; Teutsch et al. 2009).

Hand eczema (HE) is a common skin disease with 1-year period prevalence rates reportedly ranging from 6% to 11% in the general population of northern Europe (Belsito 2005; Diepgen and Coenraads 1999). Some occupations, e.g. hairdressing and nursing, show an increased risk of HE due to the frequent contact with irritants or allergens (Chew and Maibach 2003; Diepgen 2003). Hand eczema also has an endogenous genetic component (Kezic et al. 2009). Recent research findings on exposure to irritants or allergens and on markers of genetic susceptibility can be used to create a genetic test that estimates a personal relative risk for HE: a hand eczema genetic susceptibility test (de Jongh et al. 2008a, b; Molin et al. 2009). If such a test is offered to student nurses, it may contribute to the prevention of HE in this profession. The test results could be used for personal preventive measures, e.g. wearing special gloves, or even for choosing another career within or outside of the profession. It is not unlikely that such a test will be developed in the near future, especially regarding the high prevalence of HE. Currently, the predictive value of certain genetic polymorphisms for the risk of HE is under study.

The purpose of this study was to compare the output (per participant) of focus groups, interviews and questionnaires in revealing barriers and facilitators from student nurses for using a new genetic test for susceptibility to hand eczema. For this purpose, we first established the number of different items that can influence student nurses’ decision to use this new genetic test for each involvement method (output). Subsequently, we evaluated the output in relation to the number of participants needed to obtain this output.

## Methods

### Study population

The designated study population consisted of student nurses who were at least 16 years of age and attended one of three nursing schools in Amsterdam, the Netherlands. Before recruitment, the school institutional review boards agreed with the study protocol. In total, four different recruitment techniques were used. First, by e-mail, we invited 154 students who studied in the Amsterdam area and participated in an on-going national cohort study (Visser et al., unpublished data). In this national cohort of approximately 700 student nurses, genetic susceptibility towards HE is studied. Secondly, we gave 2-min introductions in classes to invite students to participate. Thirdly, we placed posters on school message boards and school cafeteria tables. Lastly, by means of convenience sampling, we approached student nurses at the schools directly. We made sure that the proportions of participants recruited with these four techniques were comparable in the focus groups, interviews and questionnaires. All recruitment methods included a brief explanation of the study and a reward for participation. When desired, participants were refunded their travel costs.

### Data collection

The execution and analysis of the three qualitative research methods were based on core literature (Bryman 2001; Denzin and Lincoln 2000; Kitzinger 1995; Kvale 1996). To create a topic list for guiding the involvement methods and the analysis of results, we first performed a literature search on factors (items) that could influence nurses’ decisions, beliefs or attitudes towards the use of a genetic test that estimates the personal risk for HE. The following search strategy was applied in MEDLINE via PubMed: (“Dermatitis, Irritant” [Mesh] OR “Dermatitis, Occupational” [Mesh]) AND (“Nurses” [Mesh]) AND (“Genetic Predisposition to Disease” [Mesh] OR “Genetic Testing” [Mesh]). Because this search did not reveal any relevant studies, we broadened the search with the following strategy: (“Genetic Predisposition to Disease” [Mesh] OR “Genetic Testing” [Mesh]) AND (“Attitude” [Mesh] OR “Public Opinion” [Mesh] OR beliefs [tw] OR facilitator [tw] OR barrier [tw]). This search was limited to information published between September first 1999 and September first 2009, to human studies and to papers published in the English language. This search revealed 1,502 possibly relevant studies. MR and MV independently scanned all retrieved citations based on title and abstracts. Subsequently, the full texts of articles of relevant abstracts were retrieved. Ten relevant studies were selected for the purpose of this investigation (Cameron et al. 2009; Cameron and Muller 2009; Condit 2001; Harel et al. 2003; Henneman et al. 2004, 2006; Sanderson et al. 2004; Sussner et al. 2009; Tercyak et al. 2006; Toiviainen et al. 2003). From these studies and from our personal experience, we formulated 22 items that could influence the use of a genetic test. The items were clustered in 10 domains and processed in a topic list (“Appendix 1”). The 10 domains were: (1) expected use of genetic test (results); (2) test content; (3) feelings and emotions; (4) involvement with HE; (5) principles/beliefs; (6) expected effects of HE; (7) relative risk of developing HE; (8) accessibility, safety and privacy; (9) practical considerations and (10) social influence and media.

**Fig. 1:**
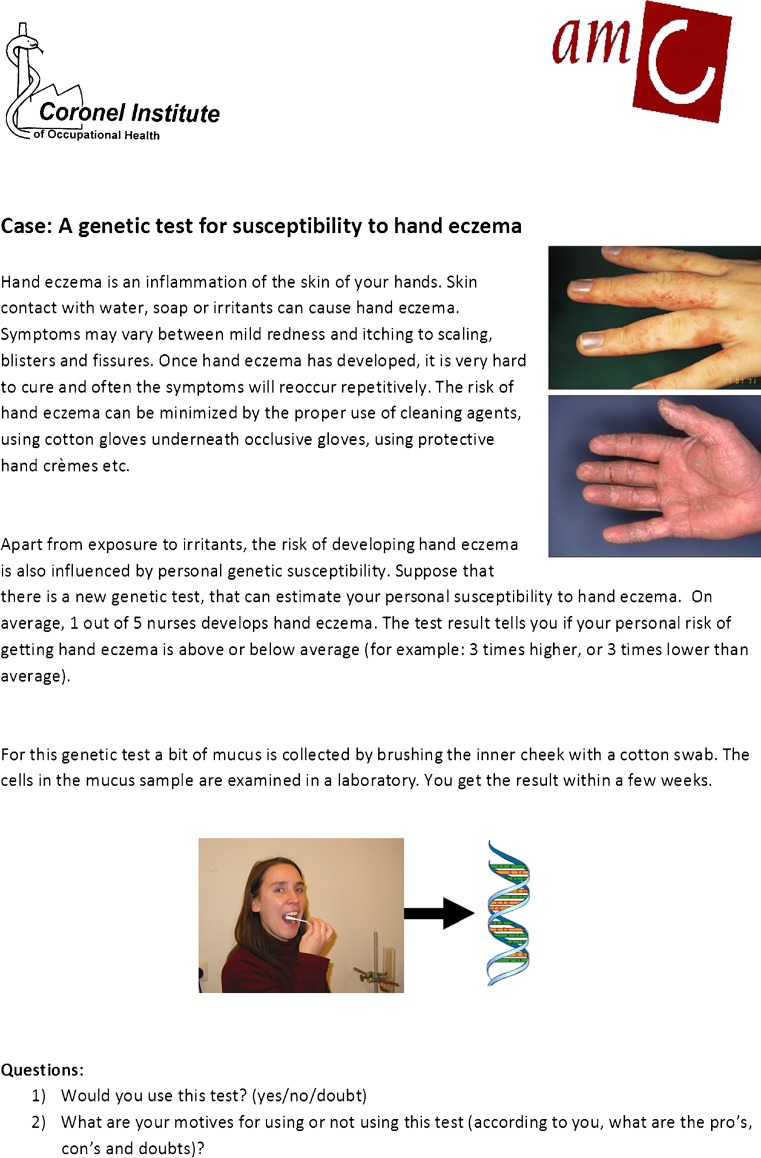
Case: a genetic test for susceptibility to hand eczema. The case was used to guide the focus groups, interviews and questionnaires

All three involvement methods comprised two parts and started with an introduction on the purpose of the study, the time schedule and confidentiality. During the first part, following the introduction, a hypothetical “case” was presented in which a genetic test for susceptibility to HE was introduced (Fig. 1). This presentation was concluded by two questions: (1) Would you use this test? (yes, no or doubt) and (2) What are your motives for using or not using this test? (open question). In the focus groups and interviews, answers were first noted by the participants and were subsequently discussed. During the second part, we introduced and discussed a topic list with items extracted from the literature. Participants were asked if (yes or no) and how (open question) the different items of this topic list would influence their choice to use this test. The items that had already been discussed during the first part were not reviewed. After this discussion, participants were invited to mention supplemental items.

Before application, the focus group protocol, interview protocol and questionnaire were all piloted. Additionally, a draft version of the electronic questionnaire was tested on comprehensibility among four workers from the Academic Medical Center in Amsterdam, the Netherlands. By convenience sampling, we recruited one worker from the catering service, one from the transport service and two student nurses.

The focus groups were held between October and December 2009 and were moderated by MR. MV participated as the case presenter and observer. Both researchers had been trained in qualitative methods. Focus group sessions lasted for about 2 h and were audio-recorded. Five to eight student nurses participated in each group, numbers depending on availability for the scheduled time. Participants received a gift coupon with a value of €20,–. The “case” was presented using PowerPoint and ended with the two discussion questions. We stimulated discussion by asking open-ended, non-guiding questions and encouraged all participants to contribute. To facilitate the discussion of the topic list in the second part of the session, we presented each domain (if not mentioned before) on flip-over sheets. We stopped the data collection at the point of data saturation, i.e. when two subsequent focus groups did not reveal any new items that could influence using a genetic test for HE.

Semi-structured interviews were executed between February and April 2010 by MR, MV and MMV. The interviews lasted for about 45 min, were audio-recorded and took place in a quiet room. Participants received a gift coupon with a value of €10,–. The “case” and the questions were provided in text and read out loud to the participants (Fig. 1). After reading the case, the interviewer left the room for a short period while the participants noted down their answers. Subsequently, the answers were discussed. To facilitate the discussion of the topic list in the second part of the interview, we presented all clustered literature items to the participants (if not mentioned before) on small cards. The interview data collection process was ended at the point of data saturation, i.e. when three subsequent interviews did not reveal any new items.

The electronic questionnaire, with combined closed and open-ended questions, was emailed to 51 participants in May 2010. We sent out one email reminder. Respondents were rewarded with a small gift (value €5,–). Participants received an introductory email with a hyperlink to the electronic questionnaire, which included 56 questions and took about 20 min to complete. The questionnaire mainly followed the protocols of the focus groups and interviews, which involved starting with the “case” and the two discussion questions on the use of the test and related motives. Subsequently, we introduced the domains one by one on separate pages. For each of the items within these domains, participants were asked if (yes or no) and how (open question) the item would influence their choice to use this test. Before proceeding to the next domain, participants were invited to provide supplemental items. Respondents were not able to go back to a previous page. The questionnaire data collection was ended at the point of data saturation, i.e. when five subsequent questionnaires did not reveal any new items.

All three methods were concluded by the participants’ completion of a short questionnaire on personal and professional characteristics and general knowledge of and experience with genetics and genetic testing (“Appendix 2”). Because we believe that the stakeholders’ perceived satisfaction with their involvement and contribution during the involvement method can directly influence their involvement output, we added the question: “How satisfied are you with your contribution during the focus group/interview/survey?” To prevent bias due to socially desirable answering, this questionnaire was completed anonymously.

### Data analysis and coding

MR and MV performed a thematic content analysis with the data from all involvement methods. The audio-taped data from the first part of the focus groups and interviews was transcribed and analysed using MAXQDA software (VERBI Software, Marburg, Germany, 2006) that facilitates with organising and presenting large quantities of qualitative data. Each relevant unit of text remark was coded according to the taxonomy of 10 domains and 22 items as extracted from the literature. Remarks that could not be coded according to our taxonomy were iteratively discussed by MR and MV, and if necessary, new items or domains were created. From this point on, “literature items” refer to items spontaneously mentioned during the first part of the involvement methods that corresponded with one of the 22 items extracted from literature. “New items” refer to items spontaneously mentioned that were additional to the literature. We also noted whether the items hindered or facilitated the use of a genetic test for hand eczema susceptibility.

The output per participant of an involvement method was calculated by the total number of items (literature + new) or the total number of relevant remarks (literature + new) obtained per method, divided by the number of participants in that method, i.e. the mean number of items or relevant remarks per participant. The total number of items revealed per method could not be compared statistically as the total number of items is related to the combined group and not to individuals. For interviews and questionnaires, the number of remarks per participant was compared using Wilcoxon’s rank-sum test. The number of remarks per participant in the focus groups could not be compared statistically with that of the interviews and questionnaires because the number of remarks was only available per focus group and not per individual.

To establish (i.e. rule out) possible differences in participant characteristics between the methods, we applied the chi-squared test for dichotomous variables, the Yates and Cochran test for ordinal variables and one-way ANOVA for continuous variables. For this purpose, we used *α* = 0.1.

## Results

### Participant characteristics

Determined by the saturation criteria, 80 student nurses participated in the three involvement methods. A total of 33 nurses in five focus groups, 15 interviews and 32 questionnaires (questionnaire response rate 63%) were needed. Table 1 summarises the participant characteristics. Ninety-four percent of the participants were female. Most participants were satisfied with their contribution during the involvement methods (mean grade ≥7.5). Fewer interview respondents would use the test (40%) in comparison to the participants from the focus groups and the questionnaire respondents (73% resp. 78%) (*p* = 0.02). The questionnaire group included more students from a high training level nursing school than the focus groups and interviews (*p* = 0.09). We did not observe any other statistically significant group differences in participant characteristics (*p* > 0.1).

**Table 1:** Participant characteristics and responses comparing focus groups, interviews and questionnaires

Participants characteristics	Focus groups (*n* = 33 participants)	Interviews (*n* = 15 participants)	Questionnaires (*n* = 32 participants)
Gender			
Female, %	94	87	63
Age			
Mean (min–max), years	21.9 (18–45)	23.6 (17–42)	22.0 (18−42)
Training level			
Medium, %	45	47	22
High, %	55	53	78
School year			
First, %	6	27	25
Second, %	15	13	25
Third, %	49	7	25
Fourth, %	30	53	25
Would you use the test?			
Yes, %	73	40	78
No, %	9	40	6
Doubt, %	18	20	16
Do you have a genetic disease yourself? Yes, %	6	13	13
Do you have a genetic disease in the family? Yes, %	36	33	47
Have you done a genetic test yourself? Yes, %	15	0	9
Has someone in your social environment done a genetic test? Yes, %	24	7	16
Have you heard or read of genetic tests before this questionnaire? Yes, %	85	87	91
Self-rated knowledge of genetics and genetic testing, scale 1–5			
Mean (min–max)	2.7 (1–5)	2.6 (1–4)	2.9 (1–5)
Satisfaction with contribution and involvement, scale 0–10			
Mean (min–max)	7.8 (4–10)	7.5 (5–10)	7.6 (5–10)

### Comparison between involvement methods

During the first part of the three involvement methods, participants made 355 remarks, which represented 35 different items that could influence using a test for susceptibility to HE (Table 2; “Appendix 1”). Sixteen of the 35 items had a facilitating effect on use, 10 had a hindering effect and nine could have both effects. Seventeen of the 35 items came forward during all three types of involvement methods. Of the 22 literature items, 21 were also spontaneously mentioned in one or more involvement methods; only one literature item, “religious beliefs”, was not mentioned spontaneously. Ten of the 21 mentioned literature items came spontaneously forward during all involvement methods or were spontaneously mentioned by more than 50% of the participants in at least one involvement method. These 10 items were “preventive measures”, “test is redundant: not decisive/definite to acquire HE”, “test message”, “curiosity”, “fear”, “need to know personal HE risk”, “have HE”, “have acquaintance with HE”, “seriousness of HE” and “effects of HE on personal work functioning” (Table 2). Of the 35 items, we considered 14 to be new in comparison to the literature. Seven of the 14 new items were mentioned during all involvement methods or were mentioned by >50% of the participants in one involvement method. These seven items were “extrapolating to take preventive measures for family or children”, “increase knowledge in general”, “selection of education or work type”, “low test effort”, “feelings of (in)security about developing HE”, “contribution to science” and “a test on HE goes too far”.

**Table 2:** Comparison of literature items and new items mentioned by student nurses during focus group sessions, interviews and questionnaires

Domains	Focus groups (*n* = 33 participants)	Interviews (*n* = 15 participants)	Questionnaires (*n* = 32 participants)
	% within method	% within method	% within method
Expected use of genetic test (results) for HE			
Preventive measures (+)^a^	>50	>50	10–50
Test is redundant: not decisive/definite to acquire HE (−)^a^	>50	>50	10–50
Extrapolating to take preventive measures for family or children (+)^b^	<10	10–50	<10
Test result will only lead to more (un)careful preventive behaviour (−)^b^	10–50	10–50	0
To increase knowledge in general (+)^b^	<10	10–50	10–50
Selection of education or work type (+/−)^b^	<10	<10	<10
Test content			
Test message (+/−)^a^	<10	10–50	10–50
Low test effort (+)^b^	10–50	10–50	10–50
Feelings and emotions			
Curiosity (+)^a^	<10	10–50	<10
Fear (−)^a^	10–50	10–50	<10
“Need” to know personal HE risk (+)^a^	10–50	10–50	10–50
Feelings of (in)security about developing HE (+/−)^b^	10–50	10–50	<10
Involvement with HE			
Interest in genetic diseases in general (+)^a^	0	10–50	0
Have HE (+)^a^	10–50	10–50	10–50
Have acquaintance with HE (+)^a^	<10	10–50	10–50
Professional involvement (+)^b^	<10	0	<10
Only for contribution to science (−)^b^	<10	10–50	10–50
Principles/beliefs			
Religious beliefs (−)^a^	0	0	0
Principally in favour of or against genetic testing (+/−)^a^	10−50	<10	0
Deterministic beliefs: will wait and see if I will get HE (−)^b^	<10	<10	0
Expected effects of HE			
Seriousness of HE (signs and symptoms) (+/−)^a^	10–50	10–50	10–50
Effects HE on personal work functioning (+)^a^	10–50	10–50	<10
Shame caused by HE (+)^a^	<10	10–50	0
Effects of HE on others in work (patients or colleagues) (+)^b^	<10	10–50	0
Effects on employers or employment (+)^b^	<10	0	0
Effect on daily life (+)^b^	0	<10	0
Relative risk of developing HE			
Cumulative incidence of HE in this nursing population, 1:5 (+)^a^	10–50	10–50	0
Low-risk HE skin type (pigmented) (−)^b^	<10	0	0
Accessibility, safety and privacy			
Insecurity surrounding the protection DNA and test results (−)^a^	10–50	10–50	0
Accessibility to test results (−)^a^	10–50	10–50	0
A test on HE goes too far (what is next?) (−)^b^	10–50	10–50	<10
Practical considerations			
Test expenses (−)^a^	<10	0	<10
Test location (+/−)^a^	0	0	<10
Social influence and media			
Opinion of acquaintances on a genetic test for HE (+/−)^a^	<10	10–50	0
Acquaintances (will) take a genetic test for HE (+/−)^a^	0	0	<10
Media forum used (+/−)^a^	0	<10	0

Items can have a facilitating (+) or hindering (−) effect on the use of a genetic test for susceptibility to hand eczema

^a^Literature items

^b^New items

Throughout the five focus groups, participants made 157 relevant remarks (4.8 remarks per participant), which represented a total of 30 different items (0.9 items per participant). In comparison, the 15 interviewees made 126 remarks (8.4 remarks per participant), which represented 29 items (1.9 items per participant), while the 32 questionnaire respondents made 72 remarks (2.3 remarks per participant), which represented 21 items (0.7 items per participant) (Tables 2 and 3). The interview participants gave significantly more relevant remarks per person than the questionnaire respondents (*p* < 0.0001). For the focus group participants, such comparison could not be calculated because data was on group level only. The total number of spontaneously mentioned items that were in addition to the items found in literature (in total 14) varied per method: the focus groups revealed 13 new items in relation to the literature, while the interviews revealed 11 and the questionnaires revealed 8.

**Table 3:** A comparison of remarks and items per person from focus group sessions, interviews and questionnaires

	Focus groups (*n* = 33 participants)	Interviews (*n* = 15 participants)	Questionnaires (*n* = 32 participants)
Total number of remarks (per person: mean, 25–75 percentile)	157 (4.8)	126 (8.4, 4–10)^a^	72 (2.3, 2–3)^a^
Total number of items (per person: mean)	30 (0.9)	29 (1.9)	21 (0.7)
Number of remarks describing items corresponding with literature (per person: mean, 25–75 percentile)	127 (3.8)	93 (6.2, 3–8)^a^	54 (1.7, 1–2)^a^
Number of items corresponding with literature (per person: mean)	17 (0.5)	18 (1.2)	13 (0.4)
Number of remarks describing new items in addition to literature (per person: mean, 25–75 percentile)	30 (0.9)	33 (2.2, 1–3)^a^	18 (0.6, 0–1)^a^
Number of new items in addition to literature (per person: mean)	13 (0.4)	11 (0.7)	8 (0.3)

Remarks and items may influence student nurses’ choice to use a genetic test for susceptibility to hand eczema

^a^25–75 percentiles could only be calculated for interviews and questionnaire as they provide data on the individual level

The influence on “others in work” and a “low risk skin type” were exclusively mentioned during the focus groups (Table 2). The “interest in genetic diseases in general” and the “media forum used” were solely mentioned during the interviews. The questionnaires did not reveal any new items that were not mentioned in the other two methods. Although several literature items were not mentioned spontaneously during a focus group, interview or questionnaire, they were all confirmed to be relevant for the use of the test during the discussion of the topic list in the second part of the involvement methods.

## Discussion

Per participant, interviews revealed most barriers and facilitators for using a new genetic test. On average, interview participants produced 1.9 items and 8.4 relevant remarks per participant, in comparison to 0.9 items and 3.8 remarks for focus group participants and 0.7 items and 1.7 remarks for questionnaire respondents. Although interviews revealed more items per participant, the total number of different items was similar to that revealed by the focus groups. Both methods were needed to reveal all different items present in the study population. In total, interviews revealed 29, focus groups 30 and questionnaires only 21 items. All three methods disclosed items that could influence the use of the test that had not been revealed by the literature search.

To our knowledge, there are only a few studies comparing the output of involvement methods (Fern 1982; Folch-Lyon et al. 1981; Kaplowitz 2000; Ward et al. 1991; Wutich et al. 2010). Kaplowitz (2000) studied the value of mangrove wetlands among residents living in Yucatan, Mexico and compared focus groups and interviews. The authors showed that the interviews revealed more different discussion topics than the focus groups, while we found that the total number of items was about equal. Fern (1982) who compared the number of unique items (ideas) regarding communication strategies or concerns on job opportunities for women suggested in focus groups and interviews concluded that focus group participants produced only 60% to 70% of the items that would have been produced in an individual interview. In our focus groups, participants produced 47% (0.9/1.9 pp) of the items of the interview participants. Unfortunately, both Kaplowitz (2000) and Fern (1982) did not study the differences and similarities of the output contents. Fern (1982) investigated the differences between interviews and questionnaires (“individuals working alone”) and between questionnaires and focus groups. They also found that interviews revealed more relevant items than questionnaires. However, in contrast to our study, the authors concluded that questionnaires revealed more relevant items than focus groups. Possibly, the complexity of our study topic (genetics and genetic testing) in comparison to the topic of the study of Fern and colleagues (job opportunities for women) could account for the observed differences. Participants in our focus groups and interviews often asked for clarification concerning genetics and genetic testing. The questionnaire participants did not have this opportunity. Clearly, complex topics are less suitable for the detection of new items through questionnaires. Furthermore, combining qualitative methods (triangulation) is mentioned to be an important criterion for finding all different opinions and views in a particular population (Bryman 2001; Denzin and Lincoln 2000; Kvale 1996). Similarly, in our study, both focus groups and interviews were needed to reveal all different items in the study population. The questionnaires did not add any items that were not already mentioned during the other two methods.

In contrast to our findings, Folch-Lyon et al. (1981), who compared the attitudes towards contraception in Mexico with focus groups and questionnaires, found no apparent differences between the attitudes (items) revealed by the two methods. Similarly, Ward et al. (1991) who compared the outputs (items) of focus groups and questionnaires of three studies on family planning also found that the outputs of both methods were highly similar. The authors concluded, however, that focus groups brought forward more in depth-information than questionnaires. Wutich et al. (2010) who compared the output (remark percentage) of these methods about sensitive topics in water-policy development concluded that the methods revealed similar remark percentages when they concerned low or moderately sensitive topics. The authors also concluded that the focus groups could reveal higher statement percentages when discussing very sensitive topics with an opportunity to exchange important information or present a solution. In our study, we discussed hand eczema, an occupational disease that is very common among nurses. Although HE is probably not a very sensitive topic, participants may consider HE to be serious, and viewing the test as an opportunity for HE prevention may have stimulated discussion. Again, the complexity of our study topic may have enlarged the difference between the output per participant of the focus groups and interviews and that of the questionnaires.

This study is one of the first comparing stakeholder involvement methods on revealing items that could influence the use of a new health-related knowledge product, such as a genetic test. Our study has several limitations. Although we carefully developed the protocols for all three involvement methods based on experience and literature, the reliability and validity of the involvement methods can be affected by the way it is conducted and evaluated. This topic needs some consideration. A limitation could be the effect of the interviewers (MR, MV and MMV) and focus group (MR) moderator on the output (Denzin and Lincoln 2000). Although they are supposed to stimulate discussion, making use of a moderator or interviewer may induce socially desirable answers from the participants. This in turn may decrease the reliability and validity of the findings. Another issue may concern participant recruitment and compensation. We tried to minimize the effects of these issues by standardization between methods, by for example, matching the recruitment technique and the amount of compensation. Also the coding process and its resulting taxonomy was a subjective process that included the interpretation of data by MR and MV. Possibly, other researchers would have preferred different domains and items. Furthermore, some items may overlap or fit in more than one domain. Nevertheless, the large differences in output (per participant) between interviews and questionnaires and between focus groups and questionnaires would most likely have remained. Another limitation concerns our method used to establish the point of data saturation and its potential influence on the output per participant. As customary, we established the point of data saturation as part of an ongoing process in data collection. Based on experience, we expected to need between four and six focus groups, between nine and 15 interviews and between 15 and 50 questionnaires to reach saturation. As a rule of thumb we used 30% of the minimum expected number, as the number of successive focus groups, interviews or questionnaires needed to indicate saturation (respectively, 1, 3 and 5). However, we chose to use two subsequent focus groups instead of one because using the output of only one subsequent focus group may be too dependent on chance. One may hypothesize that one focus group with five to eight participants has a larger impact on the output per participant than one individual interview or questionnaire. Nevertheless, a second analysis excluding the last two focus groups, three interviews and five questionnaires shows a largely similar distribution of the number of relevant remarks per participant: 7.5 for focus groups, 10.5 for interviews and 2.7 for questionnaires.

Another constraint is the observed group difference in training level and gender. The group of questionnaire respondents included more high training level student nurses (78%) than the focus groups (55%) and interview participants (53%). An expected effect of this difference is that more items and remarks would be revealed in the group with high training level nursing students because they may possibly have had more reflection on this topic. However, a subgroup analysis showed the opposite. A similar analysis on possible effects of gender on the output within the questionnaire group showed that the female respondents revealed a similar amount of items and remarks than male respondents. Next, the percentage of participants that were not willing to use the test was significantly higher for the interviews than for the focus groups and questionnaires. A more thorough inspection of data on individual level showed that not-willing interview participants, on average, revealed more remarks than the participants who were willing or were doubtful. Possibly, interview participants who were not willing to use the test had reflected more extensively on the advantages and disadvantages of the test. However, in the questionnaires, the number of remarks per participant did not show a tendency to differ among the participants who were and who were not willing to use the test. Therefore, it is not clear whether the ratio of participants willing and not willing to use the test influenced the higher number of remarks per participant. Furthermore, the specific nature of our studied research product, a genetic susceptibility test meant for a specific stakeholder group in a specific context, limits the generalisability of our study findings. Still, our findings on the output of different user involvement methods are probably useful when evaluating views of intended users to other genetic tests. We recommend that future research studies repeat our study design for different research products and tools in different contexts. Last, this study only compared the involvement methods on output per participant. Future studies could evaluate the efficiency of the involvement methods more thoroughly, by also addressing the more qualitative aspects of the output, e.g. the quality, depth or breadth, and by including all costs and benefits, e.g. time and effort spent on recruiting participants and transcribing or analysing data. Because in this study questionnaires only revealed a small part of the barriers and facilitators, time spared only using questionnaires was outweighed by the limited output. We estimate that overall, interviews seemed most efficient in terms of cost and benefits. Time spent to recruit participants was in favour of the interviews as we only needed 15 participants. Furthermore, the time needed to prepare and execute the focus groups and interviews was similar, although two researchers were needed to guide the focus groups. We estimate that the time to analyse the output was similar for both methods.

## Conclusions

We conclude that focus groups, interviews and questionnaires with intended users can all reveal a substantial number of barriers and facilitators to use a new genetic test. In this study, interviews and focus groups both revealed a higher number of items that can influence the use of the genetic test than questionnaires. Interviews and focus groups may be combined to reveal all potential barriers and facilitators in a study population. For the application of a new genetic test in practice, our findings suggest that interviews constitute the most appropriate method as the total of revealed barriers plus facilitators divided by the number of participants was highest. This conclusion may be valid for other health-related research products as well.

## Appendix 1

**Table 4:** Description of literature items and new items mentioned by student nurses during focus group sessions, interviews and questionnaires

Domain	Explanation of items
Expected use of genetic test (results) on HE	
1. Preventive measures^a^	1. Participant would use the test for taking measures to prevent the development or worsening of HE by minimising exposure or maximising skin care.
2. Test is redundant: not decisive/definite to acquire HE^a^	2. Participant would not use the test because he/she thinks it is redundant. A positive test will not mean you certainly acquire HE. A negative test does not guarantee you will not acquire HE.
3. Extrapolating to take preventive measures for family or children^b^	3. Participant would use the test because the test results indirectly provide information to family members or children, can be used to identify their susceptibility for HE and can possibly be a reason to take preventive measures.
4. Test result will only lead to more (un)careful preventive behaviour^b^	4. Participant would not use the test because he/she thinks that a negative test result will lead to un-careful preventive behaviour (not minimising exposure and not using sufficient skin care) or that a positive result can lead to overprotective preventive behaviour, jeopardising compliance to hand hygiene.
5. To increase knowledge in general^b^	5. Participant would use the test to increase knowledge in general
6. Selection of education or work type^b^	6. Participant would use the test result as advice in their choice of education or type of work.
Test content	
1. Test message^a^	7. Participant would use the test if the results contain clear and useful statements on personal HE susceptibility and tailored advice on possible preventive measures (from advice on the type and price of effective skin products and gloves to advice on strategies to reduce exposure at work).
2. Low test effort^b^	8. Participant would use the test because it takes no effort: a buccal swab is easy, fast and not painful.
Feelings and emotions	
1. Curiosity^a^	1. Participant would use the test just out of curiosity about their personal HE susceptibility
2. Fear^a^	2. Participant would not use the test because they fear their personal HE susceptibility
3. “Need” to know personal HE risk^a^	3. Participant would use the test because they feel a need to know their personal HE susceptibility
4. (In)security about developing HE^b^	4. Participant would use the test because he/she thinks that a test result would give a feeling of security, or as a confirmation of his/her own suspicions about susceptibility. Participant would not take the test if he/she thinks that it would only give rise to feelings of insecurity about if and when HE will develop (especially with a positive test result)
Involvement with HE	
1. Interest in genetic diseases in general^a^	1. Participant would use the test because he/she has an interest in genetics, genetic diseases or genetic testing in general.
2. Have HE^a^	2. Participant would use the test because he/she has HE now or has had it in the past and consequently knows how unpleasant HE can be.
3. Have acquaintance with HE^a^	3. Participant would use the test because he/she has an acquaintance with HE and knows how unpleasant HE can be.
4. Professional involvement^b^	4. Participant would use the test because he/she works in health care. He/she is nurse and, therefore, feels acquainted with health innovations.
5. Only for contribution to science^b^	5. Participant would *only* use the test to contribute to science. He/she does not want to know their test results.
Principles and beliefs	
1. Religious beliefs^a^	1. Participant would not use the test because of his/her religious beliefs.
2. Principally in favour of or against genetic testing^a^	2. Participant would not use the test because he/she is principally against genetic testing: you should not interfere with nature. Participant would use the test because he/she is principally in favour of genetic testing (these participants stated they were a bit tired of the people that are principally against genetic testing).
3. Deterministic beliefs: will wait and see if I will get HE^b^	3. Participant would not use the test because he or she believes that it cannot change the future: you just wait and see if you get HE or not.
Expected effects of HE	
1. Seriousness of HE (signs and symptoms)^a^	1. Participant would not use the test because he/she thinks (the symptoms of) HE is (are) not serious (“your hands only get red and itchy, and HE is not cancer”). Participant would use the test because he/she thinks (the symptoms of) HE is (are) serious.
2. Effects HE has on personal work functioning^a^	2. Participant would use the test because he/she thinks HE will impair his or her own work functioning. For example, pain can result in work absence.
3. Shame caused by HE^a^	3. Participant would use the test because he/she will feel ashamed of their HE.
4. Effects of HE on others in work (colleagues or patients)^b^	4. Patients may not want to be treated by a nurse with HE. Furthermore, colleagues may have to work more hours to sickness absence of a colleague with HE.
5. Effects on employers or employment^b^	5. Participant would use the test to convince his/her employer to supply products for adequate skin care and prevention. Participant believes that using the test will raise awareness about HE and indirectly lead to better work conditions.
6. Effect on daily life^b^	6. Participant would use the test because he/she thinks it can negatively affect functioning in daily life (for example, sports and dish washing).
Relative risk of developing HE	
1. Cumulative incidence of HE in this nursing population, 1:5^a^	1. Participant would use the test because of the high prevalence of HE in the nursing population.
2. Low-risk HE skin type (pigmented)^b^	2. Participant would not use the test because he/she knows that having a pigmented skin lowers the risk of getting HE.
Accessibility safety and privacy	
1. Insecurity surrounding the protection of DNA and test results^a^	1. Participant would not use the test because he/she doubts that their DNA and test results are sufficiently protected.
2. Accessibility to test results^a^	2. Participant would not use the test because he/she worries about disclosure of his/her test results to people such as family and employers.
3. A test on HE goes too far (what is next?)^b^	3. Participant would not use the test because he/she worries that in the future, a genetic test would be used to test for every single little defect and a lot of meaningless tests would be performed.
Practical considerations	
1. Test expenses^a^	1. Participant would not use the test if he/she has to pay (a high price).
2. Test location^a^	2. Participant would (not) use the test if the test will be a “self-test” that can be used at home (e.g. available in drugstore) or if the test will be performed at a general practitioner’s office or a hospital.
Social influence and media	
1. Opinion acquaintances on a genetic test for HE^a^	1. Participant would (not) use the test if the opinion of acquaintances on a genetic test for HE is negative/positive (family, friend, colleague etc.).
2. Acquaintances (will) take a genetic test for HE^a^	2. Participant would (not) use the test if an acquaintance will (not) use a genetic test for HE.
3. Media forum used^a^	3. Participant would use the test if the right media forum or channel is chosen through which the test is presented (e.g. schools, television and internet).

Items may influence student nurses’ choice to use a genetic test for susceptibility to hand eczema

^a^Items

^b^New items
